# Navigating primary and secondary immunodeficiency intersections: how to find IEI hidden within SID

**DOI:** 10.1186/s13223-025-01009-7

**Published:** 2026-05-08

**Authors:** Silvia Sánchez-Ramón, Stephen Jolles, Antonio Giovanni Solimando, Angelo Vacca

**Affiliations:** 1 Department of Clinical Immunology, Institute of Laboratory Medicine, and, IdISSC San Carlos University Clinical Hospital, Madrid, Spain; 2 Department of Immunology, Ophthalmology, and ENT, School of Medicine, Complutense University, Madrid, Spain; 3 Immunodeficiency Centre for Wales, University Hospital of Wales, Cardiff, UK; 4 Unit of Internal Medicine “Guido Baccelli”, Department of Precision and Regenerative Medicine and Ionian Area-(DiMePRe-J), University of Bari Aldo Moro, Bari, Italy

**Keywords:** Primary immunodeficiencies, Secondary immunodeficiencies, Haematological malignancies, Inborn error of immunity.

## Abstract

Primary immunodeficiencies, also known as inborn errors of immunity (IEIs), and secondary immunodeficiencies (SIDs) present a multitude of challenges for clinicians due to their overlapping clinical features and diverse underlying aetiologies. IEIs mainly arise from inherited genetic defects, while SIDs are acquired conditions. IEIs are associated with an increased risk of cancer, particularly haematological malignancies, which have been linked to SID, highlighting an area of overlap. It is being increasingly recognised that in the context of cancer, immune deficiencies initially attributed to secondary causes were in fact due to an underlying IEI. This article aims to provide a comprehensive guide for recognising the subtle, yet pivotal clues that may help identify an underlying IEI in patients with haematological malignancies. Combinations of clinical features aligned to the manifestations of IEI, laboratory markers, functional studies, IEI experienced histological assessment, and genetic studies, alongside recognition of atypical responses to therapy for autoimmune and inflammatory features of IEI, and atypical features of the malignancy and its response to therapy and recurrence, can help unmask the IEI hidden within SID. This distinction is of critical importance for patients and their families, as it alters both the treatment of the underlying IEI as well as potentially the approach to the treatment of malignancy.

## Introduction

**Fig. 1:**
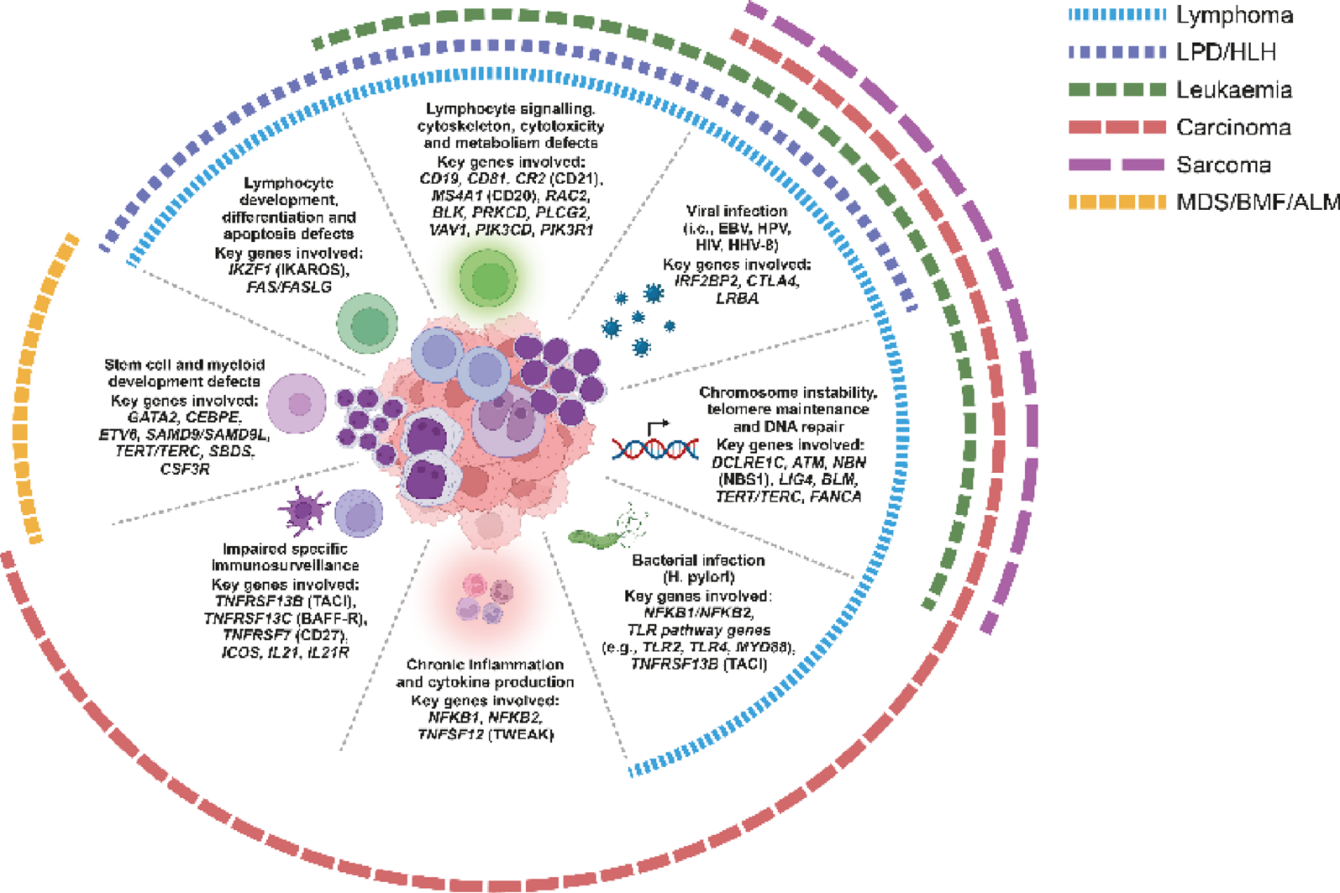
Pathogenetic pathways and genetic drivers linking IEIs to malignancy and immune dysregulation. ALM, acute leukaemia of myeloid lineage; ATM, ataxia telangiectasia mutated; BAFF-R, B-cell activating factor receptor; BLK, B lymphoid tyrosine kinase; BLM, Bloom syndrome protein; BMF, bone marrow failure; CD19, cluster of differentiation 19; CD20, cluster of differentiation 20; CD21, cluster of differentiation 21; CD27, cluster of differentiation 27; CEBPE, CCAAT/enhancer-binding protein epsilon; CR2, complement receptor type 2; CSF3R, colony stimulating factor 3 receptor; CTLA4, cytotoxic T-lymphocyte-associated protein 4; DCLRE1C, DNA cross-link repair 1 C; EBV, Epstein–Barr virus; ETV6, ETS variant transcription factor 6; FANCA, Fanconi anaemia complementation group A; FAS, Fas cell surface death receptor; FASLG, Fas ligand; GATA2, GATA binding protein 2; HHV-8, human herpesvirus 8; HIV, human immunodeficiency virus; HLH, haemophagocytic lymphohistiocytosis; H. pylori, *Helicobacter pylori*; HPV, human papilloma virus; ICOS, inducible T-cell co-stimulator; IEI, inborn errors of immunity; IKZF1, IKAROS family zinc finger 1; IL21, interleukin 21; IL21R, interleukin 21 receptor; IRF2BP2, interferon regulatory factor 2 binding protein 2; LIG4, DNA ligase IV; LPD, lymphoproliferative disorder; LRBA, lipopolysaccharide-responsive and beige-like anchor protein; MDS, myelodysplastic syndromes; MS4A1, membrane-spanning 4-domains subfamily A member 1; MYD88, myeloid differentiation primary response 88; NBN, nibrin; NBS1, Nijmegen breakage syndrome 1; NFKB1, nuclear factor kappa B subunit 1; NFKB2, nuclear factor kappa B subunit 2; PIK3CD, phosphoinositide-3-kinase catalytic subunit delta; PIK3R1, phosphoinositide-3-kinase regulatory subunit 1; PLCG2, phospholipase C gamma 2; PRKCD, protein kinase C delta; RAC2, Ras-related C3 botulinum toxin substrate 2; SAMD9, sterile alpha motif domain containing 9; SAMD9L, sterile alpha motif domain containing 9-like; SBDS, Shwachman–Bodian–Diamond syndrome protein; TACI, transmembrane activator and CAML interactor; TERC, telomerase RNA component; TERT, telomerase reverse transcriptase; TLR2, toll-like receptor 2; TLR4, toll-like receptor 4; TNFRSF13B, tumour necrosis factor receptor superfamily member 13B; TNFRSF13C, tumour necrosis factor receptor superfamily member 13 C; TNFRSF7, tumour necrosis factor receptor superfamily member 7; TNFSF12, tumour necrosis factor ligand superfamily member 12; TWEAK, TNF-like weak inducer of apoptosis; VAV1, guanine nucleotide exchange factor VAV1

Antibody deficiencies are the largest subset of immunodeficiencies and can have primary (PAD) or secondary (SAD) aetiologies [1]. Primary immunodeficiencies (PIDs), also known as inborn errors of immunity (IEI), are a group of over 500 different disorders arising from genetic defects [2, 3]. IEIs are associated with a spectrum of immune dysfunctions beyond the absence or deficiency of immune components, each contributing through distinct mechanisms—such as impaired lymphocyte development, chronic inflammation, or susceptibility to infections—to the development of specific malignancies (Fig. 1). Genetic variants cause disease, for example, by eliminating or reducing protein expression and function (null/hypomorphic) or altering the expressed protein to acquire gain-of-function features [3]. PAD comprises about 50% of IEIs [4]; however, the proportion of patients in whom antibody deficiency constitutes a component of their condition is estimated to be around 75% [5]. It has been estimated that the prevalence of PAD is around one in 2000 children, one in 1200 individuals of any age, and one in 600 households in the US [1]. IEI are largely undiagnosed and underreported, and it is estimated that between 70 and 90% of people living with an IEI are undiagnosed worldwide [6]. This delays timely and appropriate treatment and leads to poorer outcomes. Additionally, the lack of an accurate and early diagnosis of IEIs increases the risk of an incorrect diagnosis of secondary immunodeficiency (SID), as over time more patients who develop manifestations of IEI are subsequently treated with immunosuppressive drugs or chemotherapy, which can also result in SID [1, 7].

SIDs arise as a consequence of underlying diseases such as malignancy, specific treatments, transplantation, protein (antibody) loss, and infections such as human immunodeficiency virus infection, malaria, and malnutrition [1]. SAD is estimated to be 30-fold more prevalent than PAD [1].

Both SID and IEIs are associated with an increased risk of infections that can be serious [7]. SID-related infectious complications are responsible for up to 50% of deaths in patients with chronic lymphocytic leukaemia (CLL) and up to 22% and 33% of deaths of patients with multiple myeloma (MM) and non-Hodgkin lymphoma (NHL), respectively [8]. A study by Pérez et al., reported that infections account for 34.6% of deaths in patients with IEIs [9].

## Infection, immunodeficiency, and cancer predisposition

Chronic infections represent major causes of cancer. A 2012 study reported that, of 14 million new cancer cases in 2012, 15.4% were attributable to carcinogenic infections [10]. The main infectious agents were *Helicobacter pylori* (770,000 cases), human papillomavirus (HPV) (640,000), hepatitis B virus (420,000), hepatitis C virus (170,000) and Epstein–Barr virus (EBV) (120,000). It has been estimated that 5–10% of all NHL cases might be attributable to EBV [10]. Additionally, up to 90% of cancers in patients with Wiskott–Aldrich syndrome (WAS) consist of frequent EBV-positive lymphoma or leukaemia [11]. Cytomegalovirus (CMV) disease is an under-recognised complication of common variable immunodeficiency (CVID) [12]. Current evidence suggests that CMV is a plausible cancer-causing virus, as its presence has been reported in over 90% of common tumour types, while being absent in normal tissue surrounding the tumour [13]. In patients with IEIs, chronic infections, such as EBV and HPV are associated with an increased risk of cancer [7]. Conversely, cancer can lead to SID, demonstrating a bidirectional link between malignancy and immunodeficiency. Therefore, while immunodeficiency can precede the development and progression of cancer, the underlying diagnosis is often overlooked as the focus is aimed at the cancer.

Susceptibility to EBV-driven B-cell lymphoproliferation varies by IEI subgroup. In severe combined immunodeficiencies (SCID T–B+, IL2RG, JAK3, IL7RA, and CD3 subunits) and hypomorphic RAG1/2, LIG4, DCLRE1C (“leaky SCID”), EBV-associated lymphomas are uncommon—often averted by early transplantation or possibly underreported in the past due to early mortality from infections caused by other pathogens. Combined immunodeficiencies (CID; STIM1, RASGRP1, CTPS1, MST1, GATA2, DOCK8, WAS, CORO1A, PIK3CD-GoF, PIK3R1, and NF-κB1) show a spectrum of EBV-B-lymphoproliferative disorders (EBV-BLPD) risk—from rare in NF-κB1 haploinsufficiency, to > 70% in CTPS1 and RASGRP1 deficiencies—reflecting the impact of the varied impairments in T-cell proliferation, survival, synapse formation, and migration, on defence against EBV.

In contrast, selective B-cell defects (e.g. XLA, IGHM, CD79A, CD79B, IGLL1, BLNK, CD19, and CD81) rarely predispose patients to EBV-driven lymphomas. Finally, T/NK-specific IEIs—X-linked lymphoproliferative (XLP) syndromes (XLP-1, SH2D1A; XLP-2, XIAP), ITK, MAGT1, CD27, CD70, and TNFRSF9—exhibit very high penetrance of EBV-triggered lymphoproliferative disorder and lymphoma, pinpointing non-redundant roles for cytotoxic granule exocytosis and co-stimulatory pathways in EBV clearance [14, 15].

Additional IEI-associated cancer predisposition syndromes include chronic granulomatous disease (CGD), STAT1 Gain-of-Function (GOF) mutations, X-linked lymphoproliferative syndromes, and CARMIL2 deficiency. CGD is caused by defects in any of the five subunits of the nicotinamide adenine dinucleotide phosphate oxidase complex, leading to impaired reactive oxygen species–mediated pathogen clearance. Although overall cancer incidence in CGD is low, several case reports describe solid-tumour development, and Hodgkin lymphoma, in CGD patients, suggesting that chronic inflammation and defective immune surveillance may create a pro‐tumourigenic microenvironment [16, 17]. STAT1 GOF mutations lead to constitutive STAT1 phosphorylation and upregulation of interferon-stimulated genes. STAT1-GOF patients exhibit chronic mucocutaneous candidiasis and an increased incidence of EBV-driven lymphomas (e.g., non-Hodgkin lymphoma, nodular lymphocyte-predominant HL) and epithelial cancers such as oesophageal squamous cell carcinoma [18, 19]. X-linked lymphoproliferative syndromes (XLP1 and XLP2) arise from mutations in *SH2D1A* and *XIAP*, respectively. Both syndromes are characterised by “exquisite susceptibility” to EBV infection, frequently presenting with fulminant infectious mononucleosis, haemophagocytic lymphohistiocytosis, and EBV-positive lymphomas [20]. CARMIL2 deficiency (RLTPR) is a combined immunodeficiency marked by defective CD28-mediated T-cell costimulation. Case reports link homozygous *CARMIL2* mutations to EBV-associated smooth muscle tumours and lymphoproliferation, underscoring its role in tumour immune surveillance [21, 22].

It has been reported that 10% of paediatric haematological cancers are associated with congenital syndromes [7]. Children and adolescents with certain predisposing conditions, such as deficiencies in DNA repair or an IEI, are known to have an increased risk of developing NHL [23]. However, comprehensive data characterising the full spectrum of pre-existing conditions in patients with NHL are limited. A large, multinational, retrospective study involving two major childhood NHL consortia, identified, through questionnaires, 213 patients with NHL and an associated pre-existing condition [23]. These were categorised into four subgroups: (a) cancer predisposition syndromes (*n* = 124, 58%); (b) primary immunodeficiencies (*n* = 27, 13%); (c) genetic diseases without increased cancer risk (*n* = 40, 19%) and (d) non-classifiable conditions (*n* = 22, 10%). Seventy-nine of the 124 cancer predisposition syndromes involved three high-prevalence conditions, the DNA repair syndromes ataxia telangiectasia, Nijmegen breakage syndrome (NBS), and constitutional mismatch repair deficiency. Patients with pre-existing conditions experienced inferior outcomes compared with those without a pre-existing condition, and treatment-related toxicity was a significant cause of treatment failure. These findings highlight that the identification of these types of DNA repair defects should prompt an individualised approach to immunotherapy or chemotherapy and monitoring, as well as investigation for a treatment of the immunodeficiency and associated infections such as EBV. Moreover, DNA repair deficiencies and other IEIs necessitate family testing and counselling.

Multiple factors including genetic background, chronic immune activation, viral trigger, and impaired immune surveillance contribute to the spectrum of lymphomas in patients with CVID [24]. Certain CVID-like gene defects, including *TNFRSF13B*/*TACI*, predispose to B-cell dysfunction and deficiency, impairing antibody response and conferring risk of infection, autoimmunity, and lymphoproliferation through defects in B-cell tolerance and T-follicular helper cell regulation [25–27]. Additional examples of IEI genes and gene networks involved in malignancy predisposition (Table 1) include activated phosphoinositide 3-kinase (PI3K) delta syndrome (APDS), which is characterised by increased transitional B cells and senescent CD8 T cells [29]. Gain-of-function mutations in the PI3Kδ catalytic subunit p110δ (*PIK3CD*) and loss-of-function mutations in the regulatory subunit p85α (*PIK3R1*), cause APDS1 and APDS2, respectively, for which the US Food and Drug Administration (FDA)-approved targeted therapy leniolisib, is now available [29]. Hyperactive PI3K signalling is also a component of CLL, MM, and lymphoma [30–32]. Autoimmune lymphoproliferative syndrome (ALPS) due to mutations in *FAS* and *FASLG* also predisposes to lymphoma and increased double negative T-cell numbers support this diagnosis [7].

**Table 1:** General features of IEIs and indicators suggesting an underlying IEI in patients with haematological malignancy

Category	General features of IEI	Features suggestive of underlying IEI in haematological malignancy
Clinical history	- Inflammatory bowel disease - Short stature - Progression of immunodeficiency with age (paediatric patients) - Telangiectasia - Microcephaly (seen in NBS) - Growth retardation - Dysmorphic features	- Recurrent or unusually severe infections preceding malignancy onset (e.g., > 3 serious infections/year) - Persistent splenomegaly or lymphadenopathy unexplained by tumour burden - Early-onset or atypical interstitial lung disease (e.g., GLILD) in lymphoma patients - Evidence of HLH - Atypical malignancy
Laboratory findings	- Low/absent IgA levels^a^ - Low/absent IgM levels^a^ - Lack of anti-pneumococcal antibody - Poor or absent antibody responses following vaccination - Low T-cell count - Low NK cell count^b^ - Low serum free light chain	- Low/absent IgA levels^a^ - Low/absent IgM levels^a^ - Lack of anti-pneumococcal antibody - Poor or absent antibody responses following vaccination - Low T-cell count - Low NK cell count^b^ - Low serum free light chain - Presence of HAdV, HHV, HPV, EBV, and CMV in relevant clinical contexts: • HPV: when infection is severe and resistant to standard treatment • EBV and CMV: when persistent viremia is observed that is disproportionate to the degree of immunosuppression • CMV, EBV, HAdV, and HHV: fever resulting from high level viremia in patients receiving chemotherapy [28]
Family history	- Immune dysregulation, especially adult-onset inherited gain-of-function gene variants - IEI manifestations such as recurrent and severe infections, autoimmune diseases, and enteropathy	- Family history of malignancy, for example, lymphoma
Response to cancer treatment		- Unusual toxicity to cancer treatment, for example unusual or prolonged myelosuppression (e.g., delayed neutrophil recovery > 30 days) and severe, non-dose-related mucositis or organ toxicities - Therapy-resistant disease or early relapse (< 6 months) - High incidence of severe infections during induction or consolidation (> grade 3)
Tumour characteristics		- EBV-positive lymphomas in classically “EBV-naïve” hosts - Unusual histologies (e.g., smooth muscle tumour in Allo-SCT setting) - Second primary malignancies or tumours in atypical sites within 2 years of diagnosis

^a^ In the presence of normal or low/absent IgG levels

^b^ When low cell counts are inconsistent with the malignancy or the treatment of malignancy

Allo SCT, allogeneic stem cell transplantation; CMV, cytomegalovirus; EBV, Epstein–Barr virus; GLILD, granulomatous lymphocytic interstitial lung disease; HAdV, human adenovirus; HHV, human herpesvirus; HLH, haemophagocytic lymphohistiocytosis; HPV, human papillomavirus; IEI, inborn errors of immunity; IgA, immunoglobulin A; IgM, immunoglobulin M; NBS, Nijmegen breakage syndrome; NK, natural killer; SID, secondary immunodeficiency

Autoimmunity, malignancy, and immune dysregulation may often precede infectious complications and immunodeficiency in individuals with IEIs [7]. Enteropathy has also been linked to lymphoma development in patients with CVID [24, 33]. Some studies have reported that high IgM levels at the time of CVID diagnosis correlate with increased risk of haematological malignancy [33, 34], while Wehr et al. reported that low IgM levels are associated with higher risk of a lymphoid neoplasm in CVID patients [24]. Higher age at CVID diagnosis and female sex have both been linked to increased risk of lymphoma development [24, 35, 36]. It was also reported that patients with a late-onset combined immunodeficiency phenotype had a higher prevalence of lymphoma [37].

### Genetic intersections of IEIs and tumours

A growing number of cancer genes, not yet officially recognised within predisposition panels, are also germline-mutated in a number of IEIs [38]. These include *IKZF1*,* TYK2*, and *MYD88* [38]. Additionally, several well-known IEI genes have also been identified as cancer predisposition genes, including *GATA2* and *BLM* [38]. Somatic mutations in *IKZF1*, a haematopoietic zinc finger (ZF) transcription factor, have been linked to acute lymphoblastic leukaemia [38]. Germline *IKZF1* mutations within the ZF2 DNA-binding domain have been linked to an early-onset CVID. Studies have reported alterations in immunodeficiency-related genes in patients with IEI and lymphoma [39–41].

In a cohort of non-malignant lymphoproliferative disorders in children, whole exome sequencing identified an IEI genetic diagnosis in up to 62%, with clinical prognostic implications for both potential targeted therapies and haematopoietic stem cell transplantation (HSCT) [42]. Therefore, early-onset lymphoproliferation in an appropriate clinical context must be investigated for an underlying IEI.

Dedicator Of CytoKinesis 8 (*DOCK8*) deficiency, an autosomal recessive disease caused by mutations in the *DOCK8* gene, shares some manifestations of cellular and laboratory features with WAS [43]. Bi-allelic mutations in *DOCK8* were first described in 2009, leading to a combined IEI previously referred to as autosomal recessive Hyper IgE syndrome. Typical clinical manifestations include eczema, allergies, recurrent oto-sinopulmonary infections, recurrent or severe viral skin infections, and malignancy. Clinical features often get progressively worse with time, resulting in organ damage [43]. DOCK8 plays an important role in the function of natural killer (NK) cells, T cells, and in tumour immune surveillance, and DOCK8 deficiency has been associated with tumourigenesis [44]. *DOCK8* mutation and deficiency were identified in a B-cell lymphocytic leukaemia patient and an acute myeloid leukaemia (AML) patient, respectively [44]. One study demonstrated that 44% of hepatocellular carcinoma patients had low-level expression of the *DOCK8* gene in their tumour tissue, further supporting its role in cancer [45]. Importantly, germline mutations in IEI genes can alter the equilibrium phase of cancer immunoediting, a phase in which the immune system keeps the tumour in a state of functional dormancy [46]. This has also been demonstrated in immunodeficient mice, which develop more carcinogen-induced and spontaneous cancers than wild-type mice [46]. At the same time, the development of an immunosuppressive tumour microenvironment through the accumulation of somatic mutations can further drive cancer progression [46].

CVID is the most common, clinically significant IEI, presenting with a spectrum of clinical manifestations, including lymphoproliferative disorders, autoimmunity, enteropathy, malignancy, and increased susceptibility to infections. Genes that have been implicated in monogenic CVID-like syndromes include *ICOS*,* TNFRSF13B* (TACI), *TNFRSF13C* (BAFF-R), *TNFSF12* (TWEAK), *CD19*,* CD81*,* CR2* (CD21), *MS4A1* (CD20), *TNFRSF7* (CD27), *IL21*,* IL21R*,* LRBA*,* CTLA4*,* PRKCD*,* PLCG2*,* NFKB1*,* NFKB2*,* PIK3CD*,* PIK3R1*,* VAV1*,* RAC2*,* BLK*,* IKZF1* (IKAROS), and *IRF2BP2* [47].

*TACI*/*TNFRSF13B* is associated with CVID and increased risk of lymphoma. ICOS regulates T-follicular helper cell development and is associated with CVID, enteropathy, and lymphoma [48]. LRBA regulates T-cell tolerance and is linked to CVID, autoimmunity, and enteropathy [49]. *CD19* [50], *CD81* [51], and *MS4A1* [52] are all implicated in B-cell development and function. Defects lead to hypogammaglobulinaemia and increased infection risk in CVID. *CTLA4* is important for T-cell activation and tolerance, and deficiency leads to life-threatening autoimmunity, lymphadenopathy, increased lymphoma risk, and increased risk of virally driven malignancy such as EBV-associated cancers [53].

The common ground between NHL and CVID was explored in an analysis of 1,309 NHL samples, which found that 323 (25%) of the samples harboured somatic variants of genes involved in the CVID disease spectrum, half of them occurring simultaneously [54]. The most recurrent alteration present in these samples occurred in *PIK3CD* (6%) and *STAT3* (4%) [54]. The pathway analysis of common gene alterations showed enrichment in lymphoproliferation, inflammation, proliferation control (APDS [55]), immune surveillance (GATA2 haploinsufficiency [56]), and defective DNA repair mechanisms (ataxia telangiectasia [57]), similar to those affected in CVID, with *PIK3R1* emerging as a central node in the protein interaction network [54].

A systematic review and meta-analysis found that the overall prevalence of malignancy in a total of 8,123 CVID patients was 8.6% [33]. The prevalence of lymphoma and gastric cancer in these patients were 4.1% and 1.5% [33], respectively, compared with 2.1% and 0.8% in the general population [58, 59]. Clinical and laboratory clues for haematological malignancy in patients with CVID include pre-existing or concomitant lymphoproliferative disease [35]. CVID patients with lymphoproliferative disease had 2.5 times increased odds of being diagnosed with lymphoma compared with CVID patients without a lymphoproliferative disease (*p* = 0.005) [35]. CVID patients with longstanding lymphadenopathy and splenomegaly are at increased risk of being diagnosed with a lymphoid neoplasm [24].

Compared with age-adjusted cancer incidence in the Surveillance, Epidemiology and End Results Program database, patients with IEI who enrolled in the United States Immune Deficiency Network registry had up to 10-fold increased incidence of lymphoma [60]. For immunologists, this underpins the importance of awareness of the genes and IEIs that are responsible for conferring enhanced cancer predisposition. Haemato-oncologists treating young patients presenting with novel or unusual cancers, should have a heightened awareness of the possibility of an underlying IEI. All clinicians who treat patients with IEI manifestations would benefit from enhanced knowledge of the overlapping genetic susceptibilities to cancer seen in certain IEIs.

## Methods

This narrative review was conducted to gather current knowledge on the intersections between IEI and SID. Relevant articles were identified through a non-systematic search of PubMed. Keywords and search terms included “primary immunodeficiency”, “secondary immunodeficiency”, “lymphoproliferation”, “primary and secondary immunodeficiency overlap”, “inborn error of immunity”, and combinations thereof. Only articles published in English were considered. Conference abstracts and posters were excluded. Additional sources were identified through manual searches of reference lists from key articles. A total of 115 articles were evaluated.

Studies were selected based on their relevance to the review objectives. No formal quality assessment or risk of bias evaluation were performed, consistent with the narrative review methodology.

## How to find PID in SID

In addition to infectious and inflammatory complications, IEI is also associated with a range of non-infectious complications including autoimmunity, allergy, bone marrow failure, lymphoproliferation, and malignancy [7]. The initial presentation of a patient could be a cancer or autoimmune condition that may manifest years before the underlying immunodeficiency defect is eventually uncovered [61, 62]. For example, a lymphoid malignancy could precede and mask an underlying IEI, as lymphomas are the most common malignancy in patients with IEIs. Additionally, the malignancy itself, treatment of malignancy, or other manifestations may themselves cause secondary defects that further impair immunity. Therefore, when encountering conditions like atypical, recurrent or persistent infections, treatment-resistant autoimmunity, immune dysregulation, or lymphoproliferative disorders – especially at younger ages and associated with EBV – one should consider an as-yet undiagnosed IEI, even if immune biomarkers, such as immunoglobulin levels or lymphocyte subsets, appear initially normal or subtly abnormal.

Importantly, a family history and clinical re-evaluation combined with selective immunological screening could aid earlier diagnosis and avoid delays in managing both the predisposing defect as well as its manifestations. This has important implications for surveillance, genetic counselling, and prognostication. The heterogeneity of manifestations associated with IEI, along with the variable timing of presentation and range of specialties involved in the diagnosis and treatment, can create challenges in differentiating IEI from SID and further delay IEI diagnosis. Likewise, the diverse manifestations associated with IEI, which may be encountered less often in malignancy without an underlying IEI, may offer potential clues to discovering an IEI within SID. It is possible that on re-evaluation, immune deficiencies originally attributed to secondary causes (such as cancer biologics), may be due to an underlying IEI [7].

## Family history, clinical, and laboratory clues

Several potential indicators may suggest an IEI as opposed to an SID [7] (Table 1), with increased awareness of the breadth of IEI warning signs and manifestations being key [63].

A family history of immune dysregulation, especially adult-onset inherited gain-of-function gene variants [7], and a family history of IEI manifestations including recurrent and severe infections, autoimmune diseases, and enteropathy [7], are all potential indicators of an underlying IEI. A study by Hendaus et al., highlights the importance of eliciting proper family testing in saving lives of infants and children with IEIs [63]. Extensive assessment of family medical history led to the diagnosis of Omenn syndrome, a potentially fatal entity if not promptly and appropriately managed [63]. A 2019 retrospective study in Chinese children with IEIs, found that 20 children (17.8%) had a positive family history of IEIs [64].

Clinical clues pertaining to a potential IEI diagnosis include recurrent and/or severe infections years before the development of cancer [7], splenomegaly [7], autoimmune cytopenias, and polyclonal lymphadenopathy [7, 24]. The finding of granulomatous-lymphocytic interstitial lung disease (GLILD) may enrich for mutations in *KMT2D*,* CTLA4*,* LRBA*,* TACI*, and *RAG1* and is a clinical clue for an underlying IEI [65]. Early-onset dysregulation, such as inflammatory bowel disease, is a feature of some IEIs [3]. A combination of infectious and non-infectious complications characterise many IEIs [3]. Clinical clues in paediatric patients with an IEI include short stature and progression of immunodeficiency with age [7]. Other features observed include telangiectasia, microcephaly (seen in NBS), growth retardation, and dysmorphic features [3].

Atypical malignancy presentation, recurrence, or lack of response to treatment, can also indicate an underlying IEI [66]. For example, selective IgA deficiency is associated with gastrointestinal malignancies developing later in life [67]. Patients with *GATA2* mutations present with numerous diagnoses, including AML, chronic myelomonocytic leukaemia, HPV-positive tumours, and EBV-positive tumours [66]. In a patient with an underlying IEI, response to cancer treatment may be associated with increased toxicity, therapy-resistant disease, infections, second primary cancer, cancer recurrence, and unusual site of tumour development [7, 68]. These clues can help differentiate between an IEI and SID in patients with haematological malignancy.

While some cancer types and associated treatments are known to cause SAD and, therefore, may be more likely to trigger assessment of immunoglobulin levels, other cancers associated with an IEI may not be commonly thought to elicit genetic testing to identify an underlying IEI. MM, CLL, and NHL in particular are associated with both primary and secondary antibody deficiency, as these haematological malignancies are characterised by B-cell dysregulation. SAD is common in MM, occurring in up to 90% of patients with MM and in 45–83% of patients with smouldering MM at some point during the disease course [69]. There is an overall 8-fold increased risk for NHL for all IEIs [70].

Autoimmune manifestations are becoming more widely recognised as a component of various types of IEIs [71], reflecting underlying immune dysregulation. CLL has a strong genetic component, and a genome-wide association study has implicated dysregulation of immunity genes in CLL [72]. A separate study established a direct association between surface CTLA-4 and low serum IgG and IgA levels in patients with CLL, providing additional evidence of immune dysfunction in this haematological malignancy [73, 74]. Additionally it has been reported that individuals who developed CLL have a much higher incidence of prior autoimmune haemolytic anaemia (AIHA) compared with those who do not, with a study showing that AIHA carries a 3.86-fold increased risk of developing CLL [75].

Patients with cancer and laboratory evidence of immune abnormalities, in the presence or absence of infections, should be screened for IEIs using a practical, structured approach and, ideally, before therapy is initiated. This will allow treatment modification based on the underlying IEI [7]. Several laboratory findings can be used to help clinicians differentiate between an IEI and SID in patients with haematological malignancy. These include low or absent IgM and IgA levels, low anti-pneumococcal antibody with poor or absent antibody responses following vaccination, numerical or functional B-cell deficiency (inconsistent with the therapies used), low class switched memory B-cell phenotype (of non-clonal cells), undetectable kappa/lambda free light chains [76], low T-cell count, and low NK cell count [3] and persistently positive EBV PCR. It is important to note that specific biologics such as rituximab tend first to reduce IgM levels, with IgG levels being affected to a lesser degree and IgA levels generally being affected the least [77]. Therefore, low—particularly absent—IgA levels before treatment initiation, could signal an underlying IEI.

In a recent study involving 151 patients with SID associated with B-cell lymphoproliferative disorders, an artificial intelligence–derived algorithm incorporating two variables—serum kappa + lambda free light chain sum and a history of recurrent or severe infections during childhood—achieved a diagnostic accuracy of 91.8% in identifying an underlying IEI [78]. The study also highlighted distinct patterns in tumour behaviour and immune dysfunction in patients with IEIs compared to those with SID.

Several IEIs—such as ITK deficiency, MAGT1 deficiency, CD27 deficiency, and CTPS1 deficiency—are strongly associated with uncontrolled EBV infection and EBV-driven lymphoproliferative disorders, warranting the use of EBV PCR testing as a clinically relevant diagnostic approach [79].

## Genetic testing

While defining the underlying genetic defect may help to differentiate an IEI from SID in a proportion of cases, it is important to note that in most cases of IEIs, monogenic causative variants are not identified. A study investigating genetic mutations and clinical phenotypes in CVID, found that only 31% of CVID patients had an identifiable causative or associated genetic variant, leaving most patients (69%) without a known genetic cause [80]. In a separate study investigating the genetic causes of patients with IEIs in Kuwait, a population where consanguinity is very prevalent, genetic testing identified 70% of patients had a monogenic defect [81].

Primary immune regulatory disorders (PIRDs) are a group of conditions that carry a particularly high risk of malignancy [82]. IEIs such as ALPS and CTLA4 haploinsufficiency are associated with an increased risk of malignancy [82]. Some of the genes associated with haemophagocytic lymphohistiocytosis (HLH) also carry a high risk of malignancy [82]. Increasing awareness among physicians is pivotal to improve knowledge of the clinical presentations of PIRDs and optimise therapeutic outcomes, as these patients are likely to present to different specialties [82].

Newborn screening using T-cell receptor excision circles, is aimed to overcome diagnostic delays and hence the poor outcome of some severe IEIs [83]. Newborn screening can identify syndromes associated with a heightened cancer risk, such as ataxia telangiectasia, with diagnosis confirmed through subsequent genetic testing [84]. New initiatives using whole genome sequencing newborn screening for actionable conditions (many of which are IEIs), such as the Generations Study in the UK, may further shorten diagnostic delays [85]. Screening using calculated globulin also contributes to the improved early diagnosis of IEIs and SIDs, many of which do not manifest until later in life [86, 87].

The following clinical case underscores the value of genetic testing in differentiating IEI from SID in patients with overlapping clinical features. A child with X-linked lymphoproliferative syndrome, was initially treated for lymphoma and achieved remission. Subsequently, the patient presented with neurological symptoms, and investigations revealed CMV infection affecting both the peripheral nerves and central nervous system (CNS). Immunology consultation prompted genetic testing, which confirmed a diagnosis of XLP. EBV was also detected, and the patient underwent multiple therapeutic interventions. CNS imaging findings were consistent with CNS (HLH). Following multidisciplinary team discussion, HSCT was recommended.

While standard genetic testing for IEIs evaluates germline genetic changes, IEIs can arise from *de novo* mutations leading to genetic variants present in germ cells and/or somatic cells [88]. Somatic mosaicism, i.e., post-zygotic genetic alterations in DNA sequence, is emerging as an important contributor to IEIs [88]. A post-zygotic mosaic mutation in the cancer-associated gene *MAP2K1*, has been identified in a patient with immunodeficiency and RASopathy [89]. Most somatic mutations in IEIs are single nucleotide variations or small deletions/duplications in specific tissues [88]. It is highly challenging to detect these variants, as current sequencing methods do not have sufficient sensitivity and it is often not clear which cells are driving the disease [88]. It is, therefore, important to refine existing exome and genome analysis methods to efficiently identify mosaic variants, which will contribute to more precise diagnosis and treatment. The growing availability of tumour sequencing data will lead to the identification of additional genetic variants associated with immune dysregulation and add to the growing list of somatic and germline genetic conditions associated with immunodeficiency and cancer [54].

### Implications of recognition of IEI in SID

Early diagnosis of IEIs offers the opportunity to implement curative treatments such as HSCT and gene therapy, as well as targeted therapies that may reduce or prevent the risk of malignancy. Additionally, improved control of immune dysregulation, better management of specific infections, and enhanced clinical monitoring can further contribute to favourable long-term outcomes.

While the prevention and treatment of infections with antimicrobials and immunoglobulin replacement therapy (IgRT) remains central to the management of immunodeficiency, improved understanding of the pathophysiology of the inflammatory complications of IEIs has contributed to the development of mechanism-based therapeutic strategies [90]. Small molecules and biologics are effective in the treatment of clinical symptoms of specific IEIs and as a bridging therapy to HSCT [90]. Knowledge of a pre-existing IEI in patients with cancer can help guide the selection of the most appropriate treatment to avoid treatment-related toxicity. For example, patients with inherited DNA repair-deficient conditions, such as ataxia-telangiectasia and NBS, are hypersensitive to chemotherapy and radiation and therefore require adapted chemotherapy protocols and alternative treatments (e.g., immunotherapy) [91]. Additionally, early treatment of an IEI, before end organ damage has occurred, may reduce the risk of cancer [66].

## IgRT

The approach to the use of IgRT in IEI and SID is different, in that IgRT represents the standard of care for patients with IEIs and associated antibody deficiency, and therapy is often life-long [92]. In SID, both the assessment of humoral immunodeficiency and its treatment are more variable and progress is being made towards consensus on harmonised treatment strategies for these patients [69, 92]. Also, for patients with SID who receive IgRT, attempts are usually made to try to discontinue this treatment as some patients may recover their antibody production, for example, following curative therapy for NHL [92]. The accessibility to different routes of IgRT administration (intravenous, conventional subcutaneous [SCIg], manual push SCIg, prefilled syringe SCIg, and hyaluronidase facilitated) and the setting of IgRT administration (hospital or home therapy) are often different for patients with IEI versus SID [93, 94]. These are optimally selected together with the patient and might be decided based on a range of factors, including lifestyle, dose requirements, venous access, the length of time they are expected to need IgRT, and the need for other treatments, including anticancer treatments [93].

## HSCT

The use of HSCT for the treatment of severe combined immunodeficiency (SCID) and an increasing number of other types of IEIs has become the standard of care [95]. New techniques for both host conditioning and graft preparation could allow for successful stem cell transplantation while minimising toxicity [95]. HSCT is a potentially life-saving and curative treatment for many IEIs [96] and may, therefore, also prevent the development of cancer in some patients. Early identification of infants affected by SCID prior to the development of infectious complications, through newborn screening programmes, and prompt genetic testing will improve HSCT outcome [96]. In the context of malignancy with an underlying IEI, HSCT can potentially cure both diseases [96]. However, for HSCT to be considered as a therapeutic option, the treating physician must recognise the underlying IEI and have expertise in the management of IEI and therapies that bridge to HSCT. Clinical indications for HSCT in adult IEIs include bone marrow failure, lymphoma or other cancer, HLH, recurrent, persistent, or life-threatening infections (including chronic active EBV), refractory autoimmune cytopenias, refractory autoinflammation (e.g., severe colitis), and vital organ dysfunction [97, 98].

### Potential role of targeted therapies

Cancer can sometimes act as the patient’s gateway to knowledge about the existence of their underlying IEI. It is crucial for the patient to receive appropriate treatment for the management of the malignancy as well as the IEI [99]. Conventional therapy and monitoring are likely to differ for patients with IEIs and cancer.

Accurately distinguishing lymphoma from benign lymphoproliferation in patients with underlying immunodeficiencies or ALPS poses a significant clinical challenge [100]. Experienced haematopathologists with expertise in IEIs are essential to histologically characterise lymph node biopsies. Features suggestive of lymphoma over reactive lymphoproliferation include diffuse rather than nodular growth patterns, atypical lymphocyte morphology, and molecular abnormalities [35, 100]. Immunophenotyping and genetic/molecular testing may help discern clonal from polyclonal populations [100]. When clinical suspicion remains high despite ambiguous pathology, repeat biopsy for molecular testing or minimal surveillance biopsy could provide clarification [35, 100]. Multidisciplinary teams are needed for integrated clinical, laboratory, and radiological assessment. Certain entities like ALPS-associated lymphoproliferation syndromes may warrant watchful waiting over aggressive chemotherapy in ambiguous cases [100]. A thorough understanding of how IEIs and associated conditions can manifest radiologically and histologically is paramount to avoid over- or underdiagnosis of lymphoproliferative disorders and to choose the most appropriate treatment [100].

Some IEIs are caused by gene defects affecting signalling pathways directly involved in the oncogenic process, such as the constitutive activation of PI3K/protein kinase B (PI3K/AKT) in APDS, which can be targeted with specific PI3K/AKT inhibitors [99]. This highlights the need to develop functional assays that can accurately assess the activation state of pathways, such as PI3K/AKT in both non-tumour and tumour cells. Taking APDS as an example, assays evaluating phosphorylated AKT levels could help determine the degree of residual pathway activation in developed cancers. This could aid patient selection for inhibitors like leniolisib, which directly target PI3Kδ. Responses could be monitored via repeated functional pathway assessments. This individualised approach may optimise outcomes by leveraging our mechanistic understanding of the underlying genetic defect. Direct links between IEI causal genes and cancer pathways highlight the need for companion diagnostic testing to rationally apply targeted therapies when malignancies complicate disease pathogenesis. Targeted therapies provide clinicians with antitumoural therapeutic tools to treat cancers in the setting of an underlying IEI, as well as therapies to potentially reduce the risk of malignancy and bridge to definitive treatments such as HSCT [99] (Table 2). The PI3K-ẟ inhibitor leniolisib, approved for APDS by the FDA, may also find utility by reducing inflammation, lymphoproliferation, and shrinking spleen size to bridge towards HSCT in those patients requiring it [29].

 In IEI-related Hodgkin lymphoma with genetic lesions (such as *ITK3, MAGT1, RASGRP14, CD27, CD70, TNFRSF9,* and *STK4*), restoring normal immune system function is beneficial, with potentially curative options through allogeneic HSCT [121]. Refractory lymphoma may suggest the presence of underlying somatic or germline variants associated with an IEI, which could once again open new horizons for the development of novel therapeutic strategies [68]. For example, in a cohort of patients with CVID and lymphoma, programmed death ligand 1 (PD-L1) and programmed cell death protein 1 (PD1) were found to be highly expressed by tumour-infiltrating macrophages and T cells in the 12 investigated samples, suggesting that PD1/PD-L1 inhibitors could become a future targeted therapy for CVID-associated lymphomas [24].

**Table 2:** Selected therapies used in IEIs with known predisposition to haematological malignancies

Therapy Category	Agent/Modality	PID Indications	Key Evidence	References
Definitive Cellular	HSCT	CID (e.g., CD40L deficiency, RAG1/2 mutations)	Long-term cure and prevention of EBV-driven lymphomas	Burns SO & Morris EC.[101]
Definitive Cellular	Gene therapy	ADA-SCID; WAS; XLA; LAD-I; Hyper-IgM syndromes; IPEX syndrome; CGD (experimental)	Restoration of immune function; potential reduction in tumour risk	Fischer A. [2]
Targeted Small Molecules	PI3K inhibitors (leniolisib, nemiralisib, seletalisib)	Activated PI3Kδ syndrome (APDS)	Leniolisib: normalised B-cell subsets, reduced lymphoproliferation; Nemiralisib: no meaningful changes; Seletalisib: improved lymphadenopathy, lung function, etc.	Rao VK et al. [102]; Begg M et al. [103]; Diaz N et al. [104]
	Abatacept, belatacept	CTLA4 deficiency, LRBA deficiency	Reversal of autoimmunity and decreased lymphoma incidence	Kiykim A et al.[105]; Schwarz C et al.[106]
	TNF alpha inhibition	DADA2	Reduced inflammation, restored endothelial integrity	Deuitch NT et al.[107]
	JAK1/2 inhibitors	STAT1/STAT3 GoF; Interferonopathies; AIRE deficiency	Reduced immune dysregulation; improved ESR, IFN score, growth, lung lesions; reduced systemic inflammation	Fischer M et al.[108]; Li W et al.[109]; Hadjadj J et al.[110]; Hadjiyannis Y et al.[111]
	Anifrolumab	Interferonopathies	Normalised IFN-I signature; symptom relief; higher BICLA response in SLE	Kretzschmar G et al. [112]; Morand EF et al. [113]
	Sirolimus	IPEX; ALPS	Alleviated diarrhea, improved enteropathy; rebalanced DNT/Treg axis, improved cytopenias and lymphoproliferation	Ye L et al. 2024 [114]; Bindl L et al. [115]; Gu H et al. [116]; Teachey DT et al.[117]
	Anti-IL-1 (e.g., anakinra, canakinumab); MAS825	Autoinflammatory diseases; MAS	Resolved fever, reduced inflammation; rapid improvement and remission in MAS	Malcova H et al. [118]; Caorsi R et al. [119]
Monoclonal Antibodies	Anti-CD20 mAb (rituximab)	XLP1, XLP2, CD27 deficiency	Preemptive EBV B-cell depletion prevents lymphoma; improved EBV–HLH outcomes	Marsh RA et al. [120]
Supportive Immunotherapy	IVIG replacement	CVID, Hyper-IgM syndromes	Reduces infection-driven inflammation; may lower secondary malignancy risk	Chapel et al. [34]

ADA-SCID, adenosine deaminase severe combined immunodeficiency; ALPS, autoimmune lymphoproliferative syndrome; APDS, activated PI3K delta syndrome; AIRE, autoimmune regulator; BICLA, BILAG-based composite lupus assessment; CAPS, cryopyrin-associated periodic syndrome; CD, cluster of differentiation; CGD, chronic granulomatous disease; CID, combined immunodeficiency; CVID, common variable immunodeficiency; DADA2, deficiency of adenosine deaminase 2; DNT, double negative T cells; EBV, Epstein–Barr virus; ESR, erythrocyte sedimentation rate; GoF, gain of function; HLH, haemophagocytic lymphohistiocytosis; HSCT, haematopoietic stem cell transplantation; IFN, interferon; Ig, immunoglobulin; IL, interleukin; IPEX, immune dysregulation, polyendocrinopathy, enteropathy, X-linked syndrome; IVIG, intravenous immunoglobulin; JAK, Janus kinase; LAD I, leukocyte adhesion deficiency type I; MAS, macrophage activation syndrome; mAb, monoclonal antibody; MKD/HIDS, mevalonate kinase deficiency/hyper IgD syndrome; PID, primary immunodeficiency; RAG1/2, recombination activating genes 1 and 2; SLE, systemic lupus erythematosus; TNF, tumour necrosis factor; WAS, Wiskott–Aldrich syndrome; XLA, X-linked agammaglobulinaemia; XLP, X-linked lymphoproliferative disease.

## Conclusions

Despite the global underdiagnosis of IEI, SID is a much larger and growing category of immunodeficiency. This review highlights the intersections between IEI and SID, with a particular focus on haematological malignancy, in which both the underlying condition and its treatment contribute to SID. Malignancy is a recognised manifestation of IEIs and may precede the development and recognition of a significant infection burden related to more overt immunodeficiency. We describe approaches to unmask the IEI hidden within SID, which involve clinical, laboratory, and genetic analyses. The most important clue pointing to an underlying IEI, is a family history of immune dysregulation, autoimmunity, enteropathy, or recurrent infections, particularly if these are early onset. Clinical evidence, as indicated by the presence of lymphadenopathy, splenomegaly, GLILD, and autoimmunity, especially in the presence of a combination of infectious and non-infectious manifestations, is a pointer to an underlying IEI. Treatment-resistant autoimmunity and inflammation, as well as syndromal findings, may also be IEI features. Laboratory clues associated with an underlying IEI, include low or absent immunoglobulins (especially IgA), suboptimal vaccine responses, and low levels of immune cells, especially when these findings are incompatible with therapy side effects or the malignancy. The response of the malignancy to therapy, including treatment resistance, enhanced toxicity, recurrence of cancer, unusual site of recurrence, and second primary cancers, offer additional potential clues to an undisclosed IEI. These clues should lead to genetic testing for IEI gene mutations to unmask an underlying IEI diagnosis, although this remains challenging for several reasons including incomplete penetrance and determining the functional significance of variants of unknown significance. The early detection of IEI within SID is pivotal, as it enables improved management of the IEI and infections and allows some patients with specific IEIs to get access to targeted therapy. Multidisciplinary work within immunology, haemato-oncology, pathology, and genetics is needed to unmask the hidden IEI within SID and optimise patient care.

## Data Availability

No datasets were generated or analysed during the current study.

## Abbreviations

AIHA: Autoimmune haemolytic anaemia
AKB: Protein kinase B
ALPS: Autoimmune lymphoproliferative syndrome
AML: Acute myeloid leukaemia
APDS: Activated phosphoinositide 3-kinase delta syndrome
BAFF-R: B-cell-activating factor receptor
BLK: B-cell lymphocyte kinase
CANDLE: Chronic atypical neutrophilic dermatosis with lipodystrophy and elevated temperature
CAPS: Cryopyrin-associated periodic syndromes
CARMIL2: Capping protein regulator and myosin 1 linker 2
CD: Cluster of differentiation
CGD: Chronic granulomatous disease
CLL: Chronic lymphocytic leukaemia
CMV: Cytomegalovirus
CNS: Central nervous system
CTLA-4: Cytotoxic T-lymphocyte associated protein 4
CTPS1: CTP synthase 1
CVID: Common variable immunodeficiency
DIRA: Deficiency of Il-1 receptor antagonist
DOCK8: Dedicator of cytokinesis 8
EBV: Epstein–Barr virus
EBV-BLPD: EBV-associated B-lymphoproliferative disorders
ENT: Ear, nose, and throat
FCAS: Familial cold autoinflammatory syndrome
FDA: Food and Drug Administration
GATA2: Gata binding protein 2
GLILD: Granulomatous-lymphocytic interstitial lung disease
GOF: Gain-of-function
HL: Hodgkin lymphoma
HLH: Haemophagocytic lymphohistiocytosis
HPV: Human papillomavirus
HSCT: Haematopoietic stem cell transplantation
IEI: Inborn error of immunity
IgA: Immunoglobulin A
IgE: Immunoglobulin E
IgG: Immunoglobulin G
IGHM: Immunoglobulin heavy constant Mu
IGLL1: Immunoglobulin lambda-like polypeptide 1
IgM: Immunoglobulin M
IgRT: Immunoglobulin replacement therapy
IL: Interleukin
IL-1R: Interleukin-1 receptor
IL-6R: Interleukin-6 receptor
IL2RG: Interleukin-2 receptor ramma
IL7RA: Interleukin-7 receptor alpha
ITK: Il2-inducible T-cell kinase
JAK: Janus kinase
JAK3: Janus kinase 3
KREC: Kappa-deleting recombination excision circles
LIG4: DNA ligase IV
LRBA: LPS responsive beige-like anchor protein
MAGT1: Magnesium transporter 1
MM: Multiple myeloma
MST1: Macrophage stimulating 1
NBS: Nijmegen breakage syndrome
NFκB1: Nuclear factor kappa-light-chain-enhancer of activated B cells 1
NHL: Non-Hodgkin lymphoma
NK: Natural killer
PAD: Primary antibody deficiency
PCR: Polymerase chain reaction
PD-L1: Programmed death-ligand 1
PD1: Programmed cell death protein 1
PID: Primary immunodeficiency
PIK3CD-GoF: Phosphoinositide 3-kinase catalytic subunit delta – gain-of-function
PIK3R1: Phosphoinositide 3-kinase regulatory subunit 1
POMP: Proteasome maturation protein
RAG1/2: Recombination activating genes 1 and 2
RASGRP1: RAS guanyl releasing protein 1
RLTPR: RGD, LRR, and PTP domain containing protein
SAD: Secondary antibody deficiency
SAVI: Sting-associated vasculopathy with onset in infancy
SCID: Severe combined immunodeficiency
SCIg: Subcutaneous immunoglobulin
SH2D1A: SH2 domain containing 1A
SID: Secondary immunodeficiency
STAT1-GOF: Signal transducer and activator of transcription 1 – gain-of-function
STIM1: Stromal interaction molecule 1
TACI: Transmembrane activator and CAML interactor
TNF-α: Tumour necrosis factor alpha
TNFRSF9: Tumour necrosis factor receptor superfamily member 9
TWEAK: Tumour necrosis factor-related weak inducer of apoptosis
WAS: Wiskott–Aldrich syndrome
XIAP: X-linked inhibitor of apoptosis protein
XLA: X-linked agammaglobulinaemia
XLP: X-linked lymphoproliferative syndrome
XLP-1: X-linked lymphoproliferative syndrome type 1
XLP-2: X-linked lymphoproliferative syndrome type 2
